# Think Vibrio, Think Rare: Non-O1-Non-O139- *Vibrio cholerae* Bacteremia in Advanced Lung Cancer—A Case Report

**DOI:** 10.3390/tropicalmed9090224

**Published:** 2024-09-21

**Authors:** Andrea Marino, Bruno Cacopardo, Laura Villa, Adriana D’Emilio, Salvatore Piro, Giuseppe Nunnari

**Affiliations:** 1 Department of Clinical and Experimental Medicine, Unit of Infectious Diseases, ARNAS Garibaldi Hospital, University of Catania, 95122 Catania, Italy; cacopard@unict.it (B.C.); giuseppe.nunnari1@unict.it (G.N.); 2 Department of Infectious Diseases, Istituto Superiore di Sanità, 00161 Rome, Italy; laura.villa@iss.it; 3 Clinical Pathology and Clinical Molecular Biology Unit, “Garibaldi Centro” Hospital, ARNAS Garibaldi, 95122 Catania, Italy; ademilio@arnasgaribaldi.it; 4 Department of Clinical and Experimental Medicine, University of Catania, 95122 Catania, Italy; salvatore.piro@unict.it

**Keywords:** *Vibrio cholerae*, non-toxigenic *Vibrio cholerae*, non-O1, non-O139, cholera, cholera bacteremia

## Abstract

*Vibrio cholerae*, a Gram-negative bacterium, is widely known as the cause of cholera, an acute diarrheal disease. While only certain strains are capable of causing cholera, non-O1/non-O139 *V. cholerae* strains (NOVC) can lead to non-pathogenic colonization or mild illnesses such as gastroenteritis. In immunocompromised patients, however, NOVC can cause severe infections, including rare cases of bacteremia, especially in those with underlying conditions like liver disease, hematologic disorders, and malignancies. This case report presents a rare instance of NOVC bacteremia in a 71-year-old patient with advanced lung cancer, illustrating the clinical presentation, diagnostic challenges, and treatment interventions required. The patient presented with fever, asthenia, and confusion, and was found to have bacteremia caused by NOVC, confirmed through blood cultures and molecular analysis. Treatment with intravenous ceftriaxone and ciprofloxacin led to a rapid clinical improvement and resolution of the infection. This case, along with an overview of similar incidents, underscores the importance of considering NOVC in differential diagnoses for immunocompromised patients presenting with fever, and highlights the necessity of timely diagnosis and targeted antimicrobial therapy to achieve favorable outcomes.

## 1. Introduction

*Vibrio cholerae*, a Gram-negative bacillus, is primarily known as the causative agent of cholera, the most famous acute diarrheal illness, globally responsible for significant morbidity and mortality. Although only a small subset of *V. cholerae* strains possess the necessary genes to cause cholera, the rest of them, often called “non-O1/non-O139 *V. cholerae* (NOVC)”, are either non-pathogenic or asymptomatic colonizers in humans, being underestimated as human pathogens [1,2,3]. They may also cause mild, sporadic illnesses like gastroenteritis or wound infections in otherwise healthy individuals [4,5].

However, in immunocompromised individuals, NOVC could cause severe extraintestinal infections, from bacteremia to sepsis, often associated with underlying comorbidities such as liver disease, hematologic disorders, and malignancies [6].

The presence of tumors and their treatments, including chemotherapy and radiation, can disrupt normal immune function and mucosal barriers, facilitating the entry/translocation of pathogens into the bloodstream [7].

O1 and O139 serogroups are primarily responsible for epidemic outbreaks, especially in regions where cholera is endemic; in contrast, non-O1/non-O139 serogroups tend to cause sporadic infections globally [8].

The case we report describes an uncommon NOVC bacteremia in a patient with advanced lung cancer. Through this report, we aim to highlight the clinical presentation, diagnostic challenges, and therapeutic interventions necessary for managing this uncommon but severe infection in immunocompromised patients. Additionally, a literature review is conducted to provide an overview of similar cases, enhancing our understanding of this uncommon occurrence.

## 2. Case Presentation

A 71-year-old patient was admitted to the emergency department (ED) of a Sicilian hospital (Italy) with a three-day history of fever (up to 38 °C), asthenia, and confusion. The patient’s medical history included advanced lung cancer with bone and liver metastases and bilateral pleural effusion, hypertension, and benign prostatic hypertrophy.

Ten days prior to admission, he had undergone pleural drainage. He reported experiencing a self-limiting episode of diarrhea lasting five days two months earlier. He denied any recent travel and was taking alectinib as anticancer therapy.

Upon admission, he was febrile (38.5 °C) with a blood pressure of 110/60 mmHg, heart rate of 90 bpm, and oxygen saturation (SaO_2_) of 98% in room air.

Laboratory tests revealed the following: WBC count of 23,900/mm³ (92.1% neutrophils, 2.4% lymphocytes), hemoglobin (Hb) of 8.9 g/dL, platelet count of 563,000/mm³, C-reactive protein (CRP) of 12.6 mg/dL, procalcitonin of 2.04 ng/mL, creatinine of 1.17 mg/dL, gamma-glutamyl transferase (GGT) of 225 U/L, aspartate transaminase (AST) of 21 U/L, and alanine transaminase (ALT) of 25 U/L. Empiric antibiotic therapy with intravenous piperacillin/tazobactam 4.5 g every 8 h was started.

Blood cultures tested positive for *Vibrio cholerae*, identified using MALDI-TOF mass spectrometry. The culture from the pleural drainage was negative. The FilmArray gastrointestinal panel performed on a stool sample tested positive for *Vibrio cholerae*. The strain was sent to the Istituto Superiore di Sanità (ISS) labs in Italy, where agglutination tests with specific sera (polyvalent O1, Inaba/Ogawa, and O139) and polymerase chain reaction (PCR) without amplification of the ctxA gene confirmed that the strain was *Vibrio cholerae* non-O1, non-O139.

Intravenous antibiotic therapy with ceftriaxone 2 g/day and ciprofloxacin 400 mg every 12 h was initiated.

After 48 h of antibiotic therapy, the fever subsided, and lab tests showed improvement (procalcitonin 0.13 ng/mL, CRP 1.7 mg/dL, WBC 8900/mm³). Blood cultures performed after 48 h were negative, and the therapy lasted for ten days. The patient was discharged in good clinical condition.

## 3. Discussion

While *V. cholerae* is primarily associated with cholera, non-toxigenic strains can lead to severe infections, due to both patients’ predisposing conditions, such as immune impairment, and bacterial virulence characteristics [4,9,10].

Immunocompromised patients, such as those with advanced cancer, are at increased risk for invasive infections due to multiple factors, including chemotherapy and radiation therapy, which severely compromise the immune system and disrupt normal mucosal barriers, favoring microbial translocation [11,12]. The patient in our case was receiving alectinib, a tyrosine kinase (ALK and RET kinases) receptor inhibitor, as targeted therapy for lung cancer, which, while effective, can contribute to immunosuppression and increase susceptibility to infections.

Several non-oncologic conditions could enhance the risk of NOVC bacteremia, as Negri et al. highlighted, reporting a case of NOVC bacteremia and cellulitis in a diabetic Italian patient after consuming raw seafood and experiencing an insect bite on his leg during a vacation by the Adriatic Sea [13].

*V. cholerae* is a temperature-sensitive bacterium. It is easily isolated from water, shellfish, and plankton in warmer temperatures, while its presence declines in aquatic environments with lower temperatures [14,15]. These findings reflect the NOVC infection rate in different locations, as reported by Baker-Austin et al. [14] and Brehm et al. [15]. Nevertheless, Laurens et al. [11] reported a singular case, which happened in winter, of NOVC bacteremia in a diabetic patient with coronary disease, who previously reported 8 days of diarrhea after ingesting seafood [11].

The case we reported was acquired in Sicily (Italy), and the patient had not traveled outside the area. Sicily typically has a temperate climate with temperatures above 20 °C for most of the year and previous studies have already revealed the presence of *V. cholerae* (toxigenic and non-toxigenic) in seawater [16].

Few cases of NOVC bacteremia have been reported, most of them occurring in individuals with liver cirrhosis, diabetes, or other forms of immunosuppression [12,13]. In a systematic review by Deshayes et al. [17], 347 cases of NOVC bacteremia were identified, with liver cirrhosis as the most common associated condition (55%), followed by malignancy (20%), with a mortality rate of approximately 33%.

The source of infection may not be easy to detect, due to several factors: bacteremia can occur without gastrointestinal symptoms, and the bacteria may enter the bloodstream through translocation via damaged intestinal mucosa or extra-intestinal sites, even without obvious diarrhea; additionally, the environmental ubiquity of *V. cholerae* in aquatic habitats and the presence of asymptomatic carriers complicate source identification [6,18].

A report by Shanley et al. [12] described a patient, with liver cirrhosis and chronic venous insufficiency, who developed NOVC bacteremia without a clear source of infection, similar to our case where the source of the infection was not identified. The patient showed improvement with ceftriaxone plus doxycycline therapy [12].

If *V. cholerae* is isolated from a patient in a resource-rich country without a history of travel to a cholera-endemic region, it is most likely a non-O1/non-O139 strain of environmental origin. While these strains can cause serious illness in susceptible individuals, they do not pose the same public health threat as toxigenic O1/O139 *V. cholerae*.

*V. cholerae* can be detected using many non-culture-dependent diagnostic systems, which are increasingly common in hospital and commercial diagnostic laboratories. In labs still using traditional methods, *V. cholerae* grows well on standard media for blood cultures. However, isolating the microorganism from stool samples typically requires selective media, like thiosulfate–citrate–bile salts–sucrose agar, to inhibit the growth of other organisms, where *V. cholerae* appears as typical yellow colonies. Laboratories should be notified when *V. cholerae*-related diarrhea is suspected so that the appropriate media can be used. Species identification relies on standard biochemical tests [19,20]. All identified *V. cholerae* strains need further analysis to determine whether they carry cholera toxin genes and if they belong to serogroups O1 or O139. Such testing can typically be conducted in state health department laboratories. Currently, cultural and serological techniques remain the gold standard for diagnosis and enable appropriate case notification and management.

We identified *V. cholera* with MALDI-TOF performed on a positive blood culture, and then the strain was analyzed by ISS labs to rule out the presence of a toxin gene, confirming the NOVC. Unfortunately, we could assess neither the exact serotype of the NOVC strain nor the antibiotic susceptibility pattern. In addition, we have a *V. cholerae* FilmArray positive result for the patient’s stool sample, which could enforce the mucosal translocation hypothesis, due to patient’s immunosuppression status.

The treatment of *V. cholerae* bacteremia should be tailored to an antibiogram and/or to epidemiological data. Intravenous ceftriaxone is used as part of empirical parenteral therapy for NOVC bacteremia or mild extraintestinal infections, whereas dual-agent therapy, which combines a third-generation cephalosporin with fluoroquinolone or a tetracycline, is recommended for patients with severe NOVC infections [6].

Xu et al. [21] and Petsaris et al. [22] reported two cases of NOVC bacteremia in patients with liver diseases. The former article describes a case involving a novel serotype Ob5 NOVC strain, identified as ST1553, in a patient experiencing fever and mild digestive symptoms, who was successfully treated with ceftriaxone monotherapy [21]. In contrast, the second article reports a case from France, where a cirrhotic patient developed bacteremia following the consumption of undercooked seafood and was effectively treated with a combination of ceftriaxone and metronidazole, followed by ciprofloxacin plus doxycycline [22].

The combination of intravenous ceftriaxone and ciprofloxacin was effective in our case, leading to rapid clinical improvement and resolution of the infection.

## 4. Conclusions

This case highlights the potential for NOVC to cause severe systemic infections in patients with compromised immune systems, particularly those with advanced malignancies. Despite the absence of traditional risk factors such as liver cirrhosis or recent travel, the patient developed NOVC bacteremia, underscoring the need for clinicians to maintain a broad differential diagnosis when evaluating febrile illnesses in immunocompromised individuals, even in non-endemic areas, particularly in regions with warmer climates.

The use of molecular diagnostic methods played a crucial role in the early identification of the pathogen, enabling timely and effective antimicrobial therapy, which was the key to the patient’s recovery. The inability to determine the exact serotype of the NOVC strain or its antibiotic susceptibility profile limits our understanding of the pathogen’s behavior and resistance patterns, suggesting the need for enhanced laboratory capabilities. Furthermore, while the combination of ceftriaxone and ciprofloxacin proved effective, standardized treatment guidelines for NOVC infections in immunocompromised patients are still lacking. This case reinforces the importance of ongoing research into the pathogenesis, epidemiology, and optimal management of NOVC infections to better inform clinical practice and improve patient outcomes.

## Data Availability

The original contributions presented in the study are included in the article, further inquiries can be directed to the corresponding author.
